# *Cryptosporidium rubeyi* n. sp. (Apicomplexa: Cryptosporidiidae) in multiple *Spermophilus* ground squirrel species

**DOI:** 10.1016/j.ijppaw.2015.08.005

**Published:** 2015-08-24

**Authors:** Xunde Li, Maria das Graças Cabral Pereira, Royce Larsen, Chengling Xiao, Ralph Phillips, Karl Striby, Brenda McCowan, Edward R. Atwill

**Affiliations:** a Department of Population Health and Reproduction, School of Veterinary Medicine, USA; b Western Institute for Food Safety and Security, University of California, Davis 95616, USA; c University of California Cooperative Extension, San Luis Obispo County, CA 93401, USA; d University of California Cooperative Extension, Kern County, CA 93307, USA

**Keywords:** *Cryptosporidium*, *Spermophilus*, *S. beecheyi*, Ground squirrels, Genotypes, Protozoa

## Abstract

Previously we reported the unique *Cryptosporidium* sp. “c” genotype (e.g., Sbey03c, Sbey05c, Sbld05c, Sltl05c) from three species of *Spermophilus* ground squirrel (*Spermophilus beecheyi*, *Spermophilus beldingi, Spermophilus lateralis*) located throughout California, USA. This follow-up work characterizes the morphology and animal infectivity of this novel genotype as the final step in proposing it as a new species of *Cryptosporidium*. Analysis of sequences of 18S rRNA, actin, and HSP70 genes of additional *Cryptosporidium* isolates from recently sampled California ground squirrels (*S. beecheyi*) confirms the presence of the unique Sbey-c genotype in *S. beecheyi*. Phylogenetic and BLAST analysis indicates that the c-genotype in *Spermophilus* ground squirrels is distinct from *Cryptosporidium* species/genotypes from other host species currently available in GenBank. We propose to name this c-genotype found in *Spermophilus* ground squirrels as *Cryptosporidium rubeyi* n. sp. The mean size of *C. rubeyi* n. sp. oocysts is 4.67 (4.4–5.0) μm × 4.34 (4.0–5.0) μm, with a length/width index of 1.08 (n = 220). Oocysts of *C. rubeyi* n. sp. are not infectious to neonatal BALB/c mice and Holstein calves. GenBank accession numbers for *C. rubeyi* n. sp. are DQ295012, AY462233, and KM010224 for the 18S rRNA gene, KM010227 for the actin gene, and KM010229 for the HSP70 gene.

## Highlights

• *Cryptosporidium rubeyi* is a newly described species of *Cryptosporidium*.
• *C. rubeyi* is isolated from *Spermophilus* ground squirrels from California, USA.
• *C. rubeyi* demonstrates host specificity given it is not infectious for mice or calves.

## Graphical abstract

**Figure:**
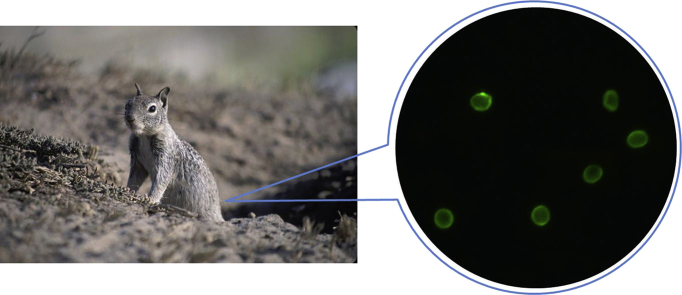

## 1 Introduction

*Cryptosporidium* spp. are a group of protozoan parasites that infect a wide range of vertebrate hosts including companion animals, livestock, wildlife, and humans. Approximately 30 species of *Cryptosporidium* have been described in vertebrate hosts that include fish, amphibians, reptiles, birds and mammals (Šlapeta, 2013). Host specificity, when documented, is highly variable between *Cryptosporidium* species, with some species or genotypes, for example *Cryptosporidium parvum*, capable of infecting multiple vertebrate hosts, while other species, for example, *Cryptosporidium andersoni*, appear restricted to a much smaller number of hosts. Systematic challenge studies for many recently described species of *Cryptosporidium* in taxonomically-related or unrelated vertebrate hosts are often lacking. Although humans and livestock are considered major biological reservoirs of a number of *Cryptosporidium* species (MacKenzie et al., 1994; Xiao and Ryan, 2004; Atwill et al., 2006; Feltus et al., 2006; Brook et al., 2009), wildlife are increasingly recognized as significant sources of environmental dissemination (Jiang et al., 2005; Feng et al., 2007; Ruecker et al., 2007; Chalmers et al., 2010) which can help foster inter-species transmission between livestock, wildlife, and humans (Hill et al., 2008; Putignani and Menichella, 2010; Raskova et al., 2013).

Ground-dwelling squirrels of the genus of *Spermophilus* are ubiquitous across California, USA. Each *Spermophilus* species inhabits a different set of ecosystems, including coastal plains and lower agricultural valleys, foothills dominated by annual grassland or oak woodlands, meadow complexes surrounded by coniferous forests, and isolated groves of pinyon pines in the remote mountains of eastern California. Colonies of ground squirrels can reach relatively high densities in suitable habitats, resulting in high rates of environmental loading of *Cryptosporidium* oocysts (Atwill et al., 2001). For example, California ground squirrels (*Spermophilus beecheyi*) can reach densities as high as 92 adults hectare^−1^ (Owings et al., 1977; Boellstorff and Owings, 1995), which when combined with shedding of up to 2 × 10^5^ oocysts animal^−1^ day^−1^ results in rates of environmental loading equivalent to 1 × 10^7^ oocysts hectare^−1^ day^−1^ (Atwill et al., 2004).

Previously we have reported a unique *Cryptosporidium* sp. c-genotype in California ground squirrels (*S. beecheyi*) (Sbey03c, 05c), Belding's ground squirrels (*Spermophilus beldingi*) (Sbld05c), and Golden mantled ground squirrels (*Spermophilus lateralis*) (Sltl05c) from throughout California, USA (Pereira et al., 2010). Based on DNA sequences of multiple genes of *Cryptosporidium*, this c-genotype is consistently different from other *Cryptosporidium* isolated from a wide range of hosts, supporting its designation as a new species of *Cryptosporidium* in *Spermophilus* ground squirrels from throughout California (Atwill et al., 2004; Pereira et al., 2010). Oocysts of Sbey03c were not infectious to neonatal BALB/c mice (Atwill et al., 2004). In the present work, we describe oocyst morphology of the c-genotype, and assess its infectivity for BALB/c mice and calves. We further characterize this genotype using 18S rRNA, actin, and HSP70 genes. The objective of the present work is to provide data on phenotypic and genotypic characteristics of c-genotype oocysts to support our assertion that this novel *Cryptosporidium* species in *Spermophilus* ground squirrels of California, USA is a new species.

## 2 Materials and methods

### 2.1 Sample collection

In 2011, 100 *S. beecheyi* squirrels from the Central Coastal region of California were sampled for additional genetic analysis of *Cryptosporidium* isolates. Squirrels were collected according to the American Veterinary Medical Association's guidelines for harvesting wildlife and feces were obtained from the large intestine and colon. Fecal samples were placed into 15 ml tubes with 5 ml of antibiotic storage solution (0.1 ml 10% Tween 20, 0.006 g Penicillin G, 0.01 g Streptomycin Sulfate, 1.0 ml amphotericin B solution, and reagent grade water for a total of 100 ml). Fecal samples were placed on ice during transportation and stored at 4 °C in the laboratory and processed within one week of collection.

### 2.2 Detection of Cryptosporidium oocysts

Detection of *Cryptosporidium* oocysts in previous studies were conducted by direct immunofluorescent microscopy (IFA) as described previously (Atwill et al., 2004; Pereira et al., 2010). Similar methods were used for the feces collected in 2011. Briefly, fecal samples were processed within one week after collection. Feces and antibiotic solution were mixed in deionized water with 0.2% Tween 20 to a final volume of 40 ml. The fecal suspension was strained through 4 layers of cotton gauze into a 50 ml centrifuge tube, which was filled with deionized water to a final volume of 50 ml. Tubes were centrifuged at 1500 g for 15 min and supernatant discarded, leaving a 1:1 ratio of pellet to solution volume. This final suspension was homogenized and 10 μl was used for making slides using the Aqua-Glo G/C Direct kit (Waterborne Inc., New Orleans, LA, USA). Slides were examined using a fluorescent microscope (Olympus BX 60) at ×400 magnification.

### 2.3 Oocyst morphology

A subset of positive fecal samples were resuspended in 40 ml of deionized water with 0.2% Tween 20 and filtered through 4-fold gauze. Filtrates were centrifuged at 1500 *g* for 10 min, supernatants discarded by aspiration, and the pellet resuspended with an equal volume of deionized water. Oocysts were purified using a discontinuous sucrose gradient method (Arrowood and Sterling, 1987) and washed 3× in deionized water with centrifuging. Oocysts were counted using a phase contrast hemacytometer and concentrations were adjusted to 10^5^ oocysts/ml deionized water and stored at 4 °C for up to 14 days before morphology was examined. Wet mount slides were prepared by pipetting 20 μl of each oocyst stock solution on to a glass slide, applying a coverslip and sealing with nail enamel. The length and width of each oocyst were measured using Nomarski Differential Interference Contrast (DIC) microscopy (Olympus BX 60) at ×1000 magnification, with an eyepiece micrometer etched with 0.2 μm divisions (reticule KR-230, Scientific Instrument Company, Napa, CA, USA). The mean length and width and the shape index (the ratio of length to width) of each isolate were calculated based on measurements of 20 intact oocysts of each isolate. These measurements were compared to the mean shape indices of 20 oocysts of *C. parvum* from a naturally infected dairy calf from central California (GenBank accession no. FJ752165).

### 2.4 Infectivity of Cryptosporidium sp. c-genotype oocysts

An *in vivo* neonatal BALB/c mouse assay (Li et al., 2010) was used to determine if *Cryptosporidium* oocysts from *Spermophilus* squirrels were infectious for this well-studied host species. Fresh oocysts were purified as described in Section 2.3 (above) and were stored in deionized water at 4 °C for approximately 3 weeks before inoculation to animals. Prior to inoculating to mice, oocysts were examined with DIC microscopy and confirmed to be intact. Female BALB/c mice with neonatal pups were purchased from Harlan Laboratories (San Diego, CA, USA), housed in cages fitted with air filters and given food and water *ad libitum*. Oocysts were administered to neonatal mice at 5 days of age by intragastric inoculation using a 24-gauge ball–point feeding needle. One hour prior to infection, the pups were removed from the dam to empty their stomachs for easier inoculation and the dam was returned to the pups after inoculation. Each litter of mice was given oocysts from only one isolate as shown in Table 2, using doses ranging from 10^2^ to10^4^ oocysts per mouse. *C. parvum* oocysts (GenBank accession no. FJ752165) purified from naturally infected California dairy calves were similarly administered to mice as a positive control, as was deionized water as a negative control. Heat inactivated (incubation at 70 °C for 2 h) *C. parvum* oocysts were also inoculated into mice to monitor pass-through of oocysts resulting from inoculation (Li et al., 2010).

*Cryptosporidium* infection in mice was assessed by staining intestinal homogenates with a FITC-labeled anti-*Cryptosporidium* immunoglobulin M antibody (Waterborne Inc., New Orleans, LA, USA) which has been shown to be a sensitive method for detecting *Cryptosporidium* oocysts from intestinal homogenates of infected mice (Hou et al., 2004). Seven days post-inoculation (PI) mice were euthanized by CO_2_ asphyxiation and the entire intestine from duodenum to rectum was collected. Intestinal samples were suspended in 5 ml of deionized water in 50 ml tubes and homogenized with an IKA^®^ Ultra-Turrax T8 tissue homogenizer (GmbH & Co. KG, Staufen, Germany). The homogenates were washed 1× in deionized water by centrifuging at 1500 *g* for 10 min and the supernatant removed. The pellets were resuspended in 10 ml of deionized water and filtered through a 20 μm pore nylon net filter (Millipore, Bedford, MA, USA) fixed on a Swinnex holder (Millipore, Bedford, MA, USA). The filtrates were concentrated to 1 ml by centrifuging at 1500 *g* for 10 min and mixed by vortexing. Fifty μl of the final homogenates were mixed with 50 μl of anti-*Cryptosporidium* monoclonal antibodies (Waterborne Inc., New Orleans, LA, USA) and 2 μl of 0.5% evans blue, then incubated at room temperature for 45 min in a dark box. Three wet mount slides were prepared from each sample using 20 μl of reaction mixture per slide. Slides were examined with a fluorescent microscope (Olympus BX 60) and a mouse was considered infected if one or more oocysts were detected in the intestinal homogenates.

In addition to the mice infectivity assay, we also conducted a trial to measure the infectivity of the c-genotype oocysts from *S. lateralis* ground squirrels in two-day old Holstein calves. Newborn calves were purchased from commercial dairy farms. For each of the eight isolates of c-genotype oocysts from *S. lateralis* ground squirrels in Tables 1 and 2, two calves were orally inoculated, one with 100 oocysts and one with 5000 oocysts. A positive control calf was given 5000 *C. parvum* oocysts from dairy calves and a negative control group of two calves were not given oocysts. Fecal excretion of *Cryptosporidium* oocysts from calves were determined using direct immunofluorescent microscopy as described above. All animal experiments with BALB/c mice and calves were approved by the Institutional Animal Care and Use Committee (IACUC) of University of California Davis.

### 2.5 Multiple gene analysis of Cryptosporidium isolates from *S. beecheyi*

Microscopic positive fecal samples were exposed to 5 cycles of freeze (−80 °C) and thaw (+70 °C) then 0.2 g was used for DNA extraction using the QIAamp DNA Stool Mini Kit (Qiagen Inc., Valencia, CA, USA) according to the manufacturer's manual. Amplification of fragments of the 18S rRNA, actin, and HSP70 genes by nested-PCR were performed using primers and cycling conditions as previously described by Xiao et al. (2000) and Jiang et al. (2005) for the 18S rRNA gene, Sulaiman et al. (2002) for the actin gene, and Sulaiman et al. (2000) for the HSP70 gene. AmpliTaq DNA polymerase (Thermo Fisher Scientific, Grand Island, NY, USA) were used for all PCR amplifications. A positive control using DNA of *C. parvum* isolated from calves from a dairy near Davis, CA as template and a negative control without DNA template were included in each PCR. PCR products were verified by electrophoresis in 2% agarose gel stained with ethidium bromide. Products of the secondary PCR were purified using the QIAamp DNA Mini Kit (Qiagen Inc., Valencia, CA, USA) according to the manufacturer's manual. Purified DNA was sequenced in both directions at the University of California DNA Sequencing Facility, using an ABI 3730 Capillary Electrophoresis Genetic Analyzer (Applied Biosystems Inc., Foster City, CA, USA).

Sequences were analyzed and consensus sequences were generated using the Vector NTI Advanced 11 software (Invitrogen, Carlsbad, CA, USA). Consensus sequences were compared to *Cryptosporidium* sequences in the GenBank using NCBI's online BLAST tool with the default algorithm parameters to target 100 sequences (http://blast.ncbi.nlm.nih.gov/) (March 12, 2015 as last day accessed). Phylogenetic analyses were conducted using Genenious Basic 5.6.5. software (Biomatters, Auckland, New Zealand). Phylogenetic relationships were inferred by using the neighbor-joining method and the Tamura-Nei genetic distance model with bootstrapping of 1000 replicates for the three genes. Depending on the availability of sequences in the GenBank, reference sequences for constructing the phylogenetic trees were selected based on: 1) sequences representing well described *Cryptosporidium* species (exclude synonyms) from fish, amphibians, reptiles, birds, and mammals, 2) sequences previously used by others for species description or as reference sequences, 3) sequence length (longer sequence if full sequence not available for each species; i.e. 18S rRNA gene sequences ≥700 bp, actin gene sequences ≥750, and HSP70 gene sequences ≥1700 bp), 4) sequences not originating from cloned PCR products due to the potential for erroneous sequence data generated from cloning PCR products (Zhou et al., 2003; Ruecker et al., 2011), and 5) previously published c-genotypes from ground squirrels (i.e. Sbey-c, Sbld-c, and Sltl-c genotypes of the 18S rRNA gene). Names and GenBank accession numbers of selected references sequences are shown in Figs. 2–4. The DNA sequences of 18S rRNA gene (GQ899206), actin gene (XM_003879845), and HSP70 gene (XM_003883591) of *Neospora caninum* were used as out-groups for constructing the phylogenetic tress. Sequences from *S. beecheyi* and all selected reference sequences were trimmed at both the 5′ and 3′ ends after alignment to use the same length for phylogenetic tree construction.

### 2.6 Statistical analysis

The mean length and mean width of oocysts from ground squirrel isolates were compared to those of *C. parvum* oocysts from a dairy calf using a two sample T-test and the SPSS Statistics 19 software (North Castle, NY, USA).

## 3 Results and discussion

### 3.1 Oocyst morphology

**Fig. 1:**
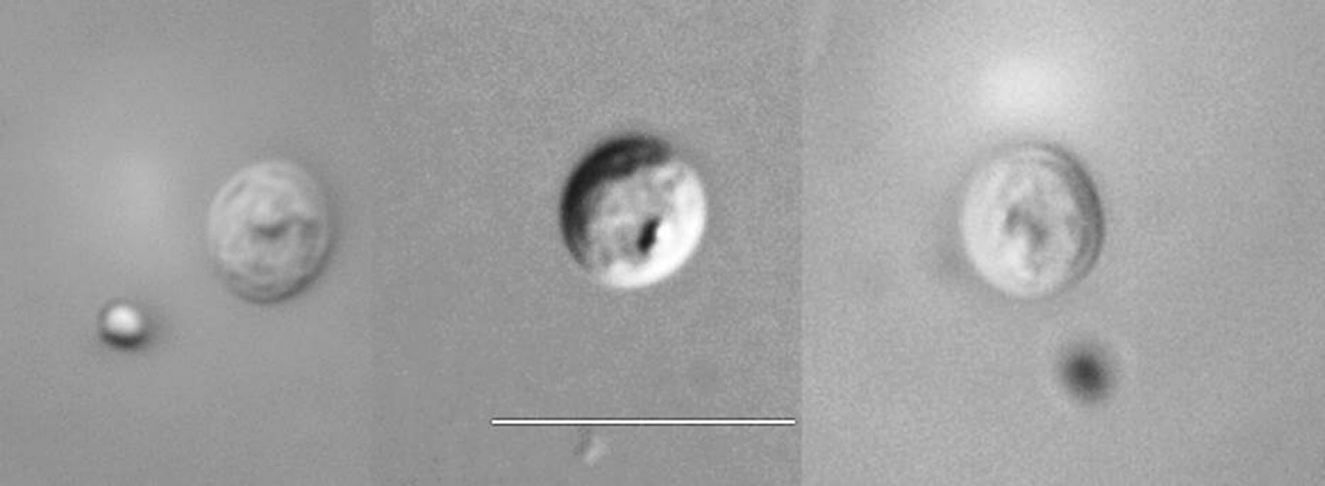
*Cryptosporidium* sp. Sbey11c oocysts from California ground squirrels (*S*. *beecheyi*). Differential interference contrast (DIC) microscopy (1000×), bar = 10 μm.

Oocysts of c-genotype from the three ground squirrel species appeared spherical or ovoid, morphologically similar to *C. parvum* oocysts from California dairy calves. The mean (±SD) size and shape index of the isolates of oocysts from *S. lateralis*, *S. beecheyi*, and *S. beldingi* are shown in Table 1. The width for all oocysts from *S. lateralis*, *S. beecheyi*, and *S. beldingi* ground squirrels were narrower than that of *C. parvum* oocysts while the lengths of the majority of isolates from all three ground squirrel species were shorter than that of *C. parvum* oocysts (Table 1). No significant differences in oocyst size were observed among c-genotype oocysts within and between each squirrel species. Mean size were 4.67 (4.4–5.0) × 4.33 (4.0–4.8) μm with a length/width index of 1.08 for oocysts (n = 160) from *S. lateralis*; 4.69 (4.4–5.0) × 4.42 (4.2–4.6) μm with a length/width index of 1.06 for oocysts (n = 40) from *S. beecheyi*; and 4.68 (4.4–5.0) × 4.27 (4.0–5.0) μm with a length/width index of 1.10 for oocysts (n = 20) from *S. beldingi*. Overall mean size of oocysts from the three ground squirrel species were 4.67 (4.4–5.0) × 4.34 (4.0–5.0) μm with a length/width index of 1.08 (n = 220). Representative differential interference contrast (DIC) photos of Sbey-c (Sbey11c) genotype oocysts from *S. beecheyi* collected in 2011 are shown in Fig. 1. The width and length of oocysts from the majority of *Cryptosporidium* species or genotypes measure between 4 and 6 microns, appear nearly spherical and have obscure internal structures (Fayer et al., 2000), thus very limited morphological characteristics are available for differentiating *Cryptosporidium* oocysts at the species or genotype level. Although oocyst morphology alone is not reliable for identifying *Cryptosporidium* species or genotypes (Fall et al., 2003), morphological analysis is an important complement to molecular and biological analysis in delineating species or genotypes of *Cryptosporidium* (Xiao et al., 2004).

### 3.2 Oocyst infectivity of Cryptosporidium sp. c-genotype

Using *C. parvum* from dairy calves as a positive control, 83% (5/6) of neonatal BALB/c mice were infected after inoculation of 100 oocysts and 100% (19/19 and 17/17) after inoculation of 5000 and 10,000 oocysts, respectively (Table 2). In contrast, oocysts of all isolates of *Cryptosporidium* sp. c-genotype from the three ground squirrel species failed to produce detectable levels of infection in mice. *Cryptosporidium* oocyst infectivity in mice varies with species and genotype, inoculum size, mouse species and strain, age, and susceptibility (Finch et al., 1993; Neumann et al., 2000; Hou et al., 2004). It is well established that neonatal BALB/c mice are susceptible to *C. parvum* infection (Fayer, 1995; Slifko et al., 2002; Jenkins et al., 2003; Guk et al., 2004). Tarazona et al. (1998) reported that inoculation of 10^4^ or more *C. parvum* oocysts results in 100% infection in BALB/c mice. We determined previously that the 50% infective dose (ID_50_) for *C. parvum* in neonatal BALB/c mice was 70.6 oocysts (Li et al., 2005) and mice inoculated with 1000 oocysts resulted in 100% infection (Li et al., 2010). Previously we have shown that inoculation up to 10^4^ Sbey03c oocysts failed to infect neonatal BALB/c mice (Atwill et al., 2004) and the current results confirm this, indicating that *Cryptosporidium* sp. c-genotype oocysts from *Spermophilus* ground squirrels are not infectious to neonatal BALB/c mice-and also exhibit some degree of host specificity. In a similar study, inoculation of 10^3^*Cryptosporidium* oocysts from red squirrels (*Sciurus vulgaris*) failed to generate detectable infection in neonatal and adult CD-1 and BABL/c mice (Kvác et al., 2008).

Intestinal homogenates coupled with fluorescent microscopy for determining *C. parvum* infection in neonatal mice has been shown to be significantly more sensitive than histopathology (Hou et al., 2004). In the present work, no oocysts were detected from mice inoculated with heat inactivated *C. parvum* oocysts, which confirmed that oocysts detected in positive control mice were not from direct inoculation and subsequent pass through but instead from patent intestinal infections. No clinical signs of cryptosporidiosis were observed in *C. parvum* infected mice which is not unusual given that asymptomatic cryptosporidial infections in mice have been documented previously by other investigators (Tarazona et al., 1998; Kvác et al., 2008). Prepatent periods of *Cryptosporidium* infection in mice vary with species and doses of oocysts, species, age, and susceptibility of mice, with younger mice generally more susceptible (Youssef et al., 1992; Tarazona et al., 1998; Matsui et al., 1999; Rhee et al., 1999; Yang et al., 2000). In the present work most mice were euthanized at day 7 PI for detection of oocysts, which was appropriate for the detection of *C. parvum* infection in mice in the present and previous work (Hou et al., 2004). To explore the possibility of a longer prepatent period for *Cryptosporidium* infection in mice from inoculation of *Spermophilus* ground squirrel oocysts, we postponed euthanasia to day 10 PI in some mice inoculated with oocysts from *S. lateralis* (isolates 113, 128, 155, and 230). Despite this longer period, no oocysts were detected in this cohort of mice. This suggests that the failure to detect *Cryptosporidium* infection in neonatal BALB/c mice was due to host specificity of *Spermophilus*-derived *Cryptosporidium* rather than the length of the prepatent period.

Although only two calves were inoculated for each of the 8 isolates, we did not find evidence of infection in calves from inoculation with up to 5000 oocysts of the c-genotype from eight *S. lateralis* ground squirrels; rather, calves in all groups including the negative control group (without oocyst inoculation) eventually became infected with *Cryptosporidium* oocysts that were confirmed to be 100% identical to the *C. parvum* via sequencing the 18S rRNA gene. This genotype of oocyst was the same as found in our positive control calf whereby the oocysts were collected from a local dairy in the same region where the calves were purchased (data not shown). Given that *Cryptosporidium* remain genetically stable after passing through mammalian species (Akiyoshi et al., 2002), these calfhood infections with *C. parvum* might be due to natural infection before inoculation or cross contamination from, for example, filth flies from nearby commercial dairies and/or from our positive control calves. Our calf pens were in an outdoor open facility which can allow filth flies to circulate between positive control and other calves. Our results of BALB/c mice and calf infectivity studies suggest there exists host specificity for this specific c-genotype *Cryptosporidium* shed by *Spermophilus* ground squirrels.

### 3.3 *Multiple gene analysis of* Cryptosporidium *sp. c-genotype isolates from* S. beecheyi

**Fig. 2:**
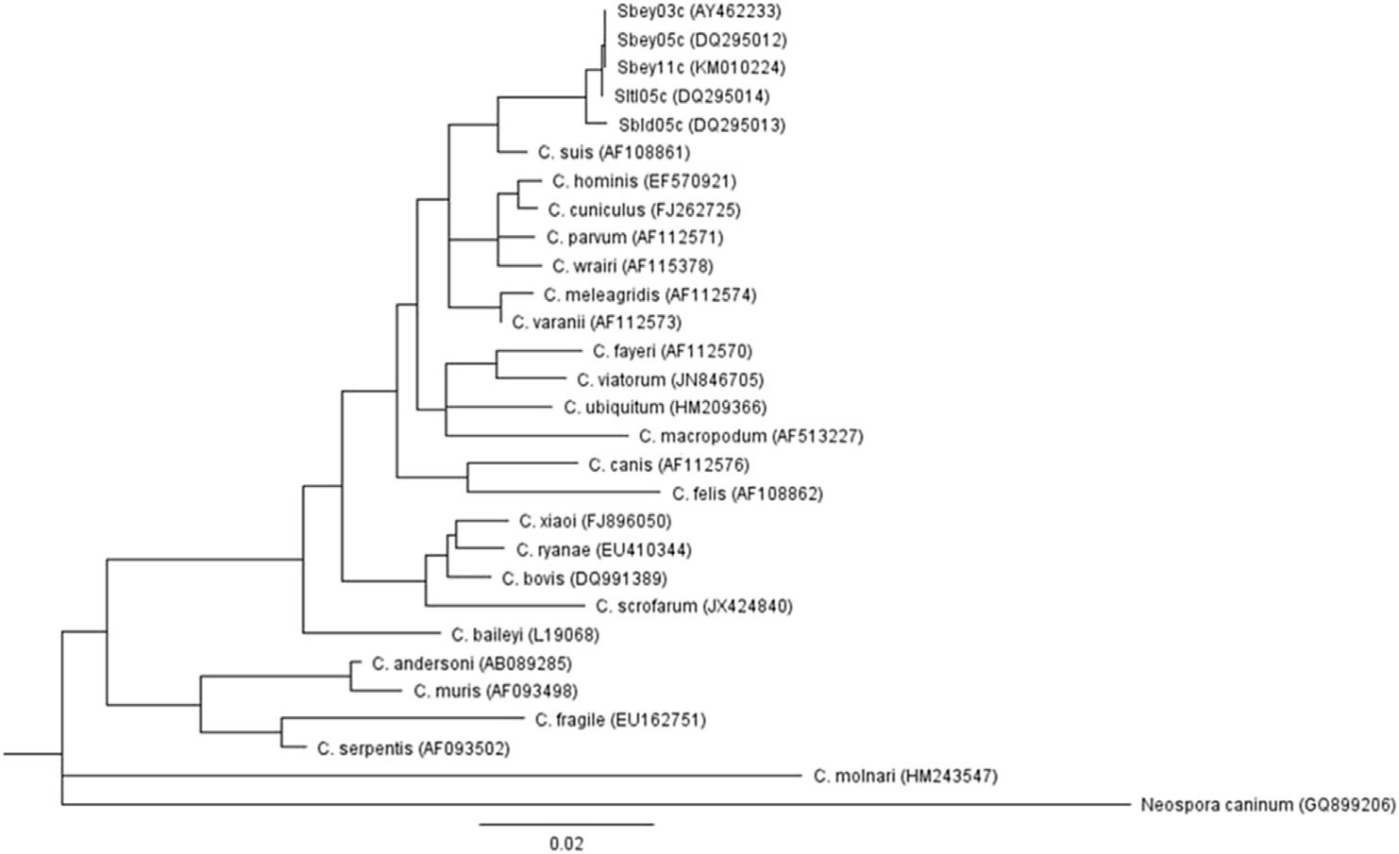
Phylogenetic relationships of partial 18S rRNA gene sequences of *Cryptosporidium* sp. Sbey11c from California ground squirrels (*S*. *beecheyi*) and other *Cryptosporidium* spp. inferred by neighbor-joining analysis with 1000 bootstrapping replicates.

**Fig. 3:**
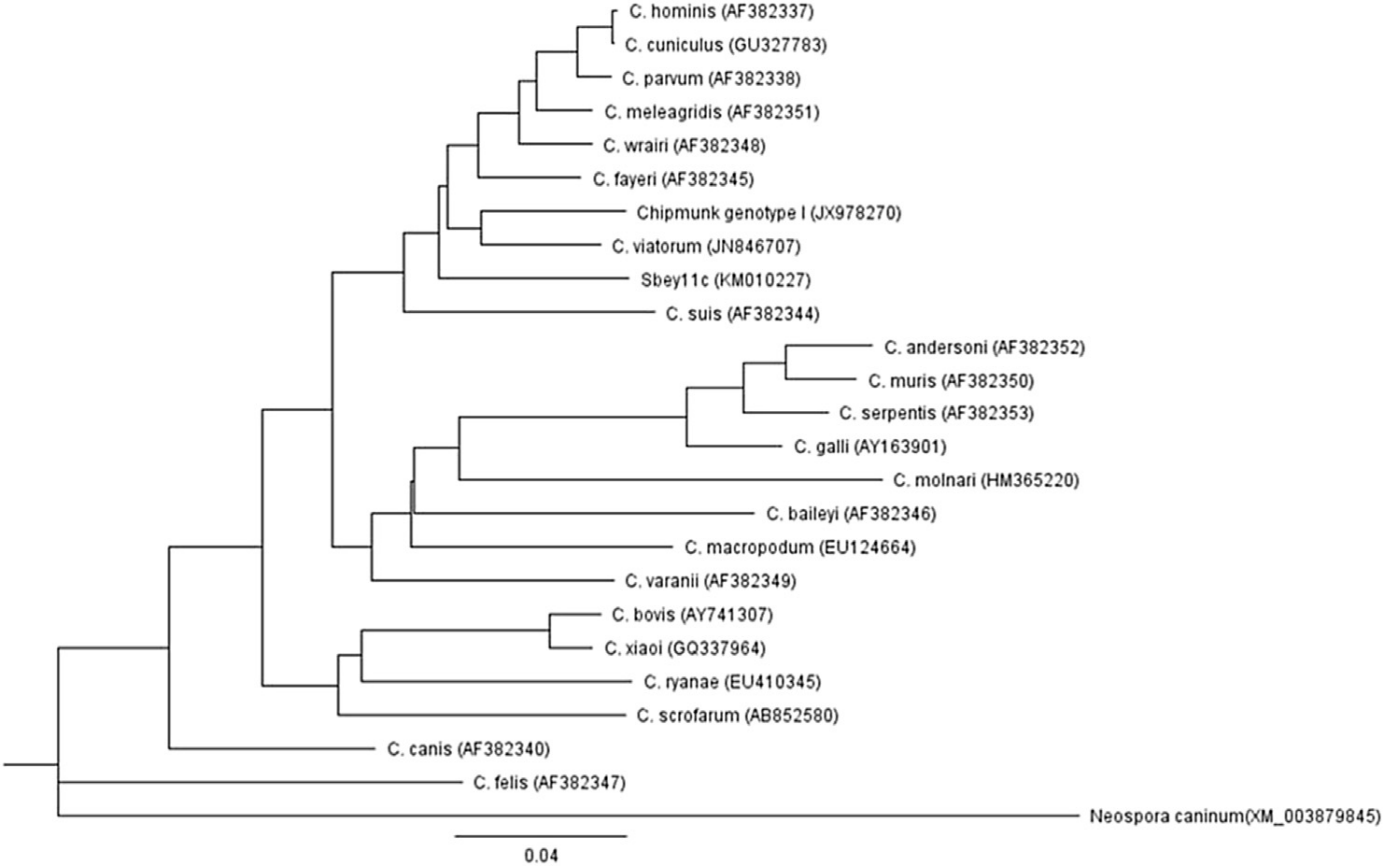
Phylogenetic relationships of partial actin gene sequences of *Cryptosporidium* sp. Sbey11c from California ground squirrels (*S*. *beecheyi*) and other *Cryptosporidium* spp. inferred by neighbor-joining analysis with 1000 bootstrapping replicates.

**Fig. 4:**
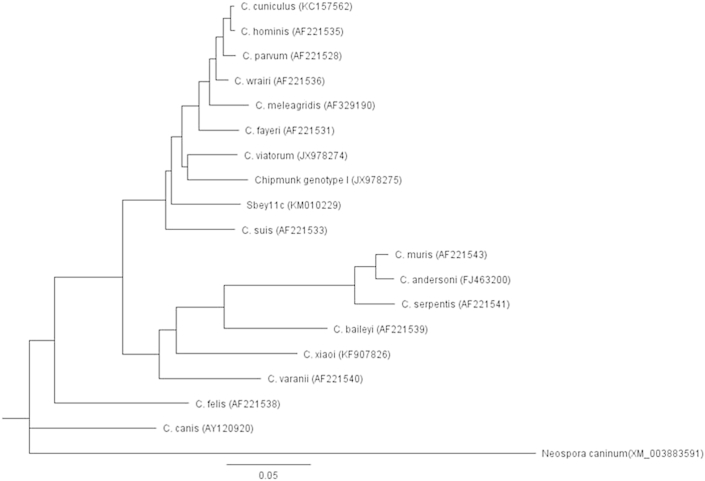
Phylogenetic relationships of partial HSP70 gene sequences of *Cryptosporidium* sp. Sbey11c from California ground squirrels (*S*. *beecheyi*) and other *Cryptosporidium* spp. inferred by neighbor-joining analysis with 1000 bootstrapping replicates.

We previously reported DNA fingerprinting of *Cryptosporidium* isolates from *Spermophilus* ground squirrels collected throughout California, USA (longitude of 114° 8′ W to 124° 24′ W and latitude of 32° 30′ N to 42° N) (Pereira et al., 2010). In this present work additional fingerprinting using 18S rRNA, actin, and HSP70 genes was conducted on new *Cryptosporidium* isolates from *S. beecheyi* collected in 2011 from the Central Coastal region of California (e.g., latitude of 35°16′N and longitude: 120°39′W) to confirm our earlier findings of a new species of *Cryptosporidium* in this host species. Among the 100 *S. beecheyi* squirrel fecal samples (each from a different squirrel), 18, 14, and 3 fecal samples with oocysts were successfully sequenced for the c-genotype by using the 18S rRNA, actin, and HSP70 gene, respectively. Using our previous nomenclature based on host species, year of isolation, and genotype, in this manuscript we describe the c-genotype collected in 2011 as Sbey11c (host *S. beecheyi*, 2011 isolation, genotype-c). According to electrophoresis and DNA sequencing results, no positive squirrels were found to be shedding more than one genotype at a time. The GenBank accession numbers of representative c-genotype sequences are KM010224 of the 18S rRNA gene, KM010227 of the actin gene, and KM010229 of the HSP70 gene, respectively. Given the small amount of fecal sample obtained using trap and release procedures for squirrels, it can be difficult to have sufficient oocysts to successfully complete PCR and multiple gene sequencing from a single isolate from this host species, but one isolate of c-genotype was successfully sequenced for all three genes. Phylogenetic trees based on DNA sequences representing the c-genotype of the three genes were constructed and juxtaposed against reference sequences of *Cryptosporidium* species/genotypes selected as mentioned above (Figs. 2–4).

BLAST results (as of March 12, 2015) of DNA sequences of the three genes are shown in Table 3. With respect to the actin gene, the Sbey11c (KM010227) was not 100% identical to any *Cryptosporidium* sequence in the GenBank, with maximal similarity of only ∼93% to a *Cryptosporidium* sp. chipmunk genotype I (JX978270). Phylogenetic analysis of the actin gene sequences revealed similar results as the BLAST analysis in that Sbey11c did not form a distinct clade with any existing *Cryptosporidium* sequence in GenBank (Fig. 3). For the HSP70 gene, maximal similarity of Sbey11c (KM010229) to currently available sequences was at best only ∼92% similar to two isolates of *Cryptosporidium* sp. chipmunk genotype I (JX978275, JX978276). Similarly, the phylogenetic analysis of the HSP70 gene shows that Sbey11c did not form a distinct clade with any existing *Cryptosporidium* sequences (Fig. 4).

For the 18S rRNA gene, BLAST results show that the Sbey11c (KM010224) was 100% identical to Sbey05c (DQ295012) and Sbey03c (AY462233), 99.64% similar to Sltl05c (DQ295014), and 98.67% similar to Sbld05c (DQ295013) (Table 3). Sbey05c and Sbey03c represent for the most common *Cryptosporidium* c-genotype from *S. beecheyi* squirrels collected in 2005 and 2003; Sltl05c represents the typical *Cryptosporidium* sp. c-genotype from *S. lateralis* squirrels collected in 2005; Sbld05c represents the typical *Cryptosporidium* sp. c-genotype from *S. beldingi* squirrels collected in 2005, as previously reported (Pereira et al., 2010). It is interesting that additional fingerprinting of new isolates collected in 2011consistently confirm the presence of Sbey-c genotype *Cryptosporidium* in *S. beecheyi*. Phylogenetic analysis of the 18S rRNA gene sequences revealed similar results as the BLAST analysis. The Sbey11c formed a distinct clade with *Cryptosporidium* isolated from all three host species (*S. beecheyi, S. lateralis, S. beldingi*) (Sbey05c, Sbey03c, Sltl05c, Sbld05c) compared to existing *Cryptosporidium* sequences (Fig. 2). In particular, BLAST and phylogenetic analyses of 18S rRNA, actin, and HSP70 genes sequences demonstrated that *S. beecheyi* are a mammalian host of the Sbey11c genotype, and that this unique genotype is also present in other species of the genus *Spermophilus* from throughout California, USA.

Focusing on the 18S rRNA gene that is commonly used for *Cryptosporidium* speciation (Checkley et al., 2015) and has the most sequences of described species and genotypes in the GenBank, our work over a decade (Atwill et al., 2001, 2004; Pereira et al., 2010 current work) has demonstrated that *Cryptosporidium* sp. c-genotype is the most prevalent genotype in *Spermophilus* ground squirrels. Combining the evidence of the presence of novel *Cryptosporidium* species in *Spermophilus* ground squirrels from our previous (Atwill et al., 2004; Pereira et al., 2010) and current work, we propose to name the *Cryptosporidium* sp. c-genotype in *Spermophilus* ground squirrels as *Cryptosporidium rubeyi* n. sp. GenBank accession numbers of DNA sequences for *C. rubeyi* n. sp. are DQ295012, AY462233, and KM010224 for the 18S rRNA gene, KM010227 for the actin gene, and KM010229 for the HSP70 gene.

## 4 Description

*Order*: Eucoccidiorida.

*Family*: Cryptosporidiidae.

*Species*: *C. rubeyi* n. sp.

*Diagnosis*: Oocysts are shed in feces fully sporulated. Oocysts measure 4.4–5.0 μm (mean = 4.67) × 4.0–5.0 μm (mean = 4.34) with a mean length/width index of 1.08 (n = 220). Prepatent period, patent period and endogenous stages are unknown.

*Type host*: California ground squirrel (*S. beecheyi*)

*Other hosts*: Belding's ground squirrel (*S. beldingi*), Golden Mantled ground squirrel (*S. lateralis*)

*Type locality*: California.

*Materials deposited*: Pending.

*Etymology*: This species name is derived from the nickname *Rube* which was given to the late father of Dr. Edward R. Atwill, School of Veterinary Medicine, University of California, Davis.

Earlier work has documented *Cryptosporidium* infections in a gray squirrel (Sundberg and Ryan, 1982), fox squirrels (Current, 1989), flying squirrels (Current, 1989), and a 13-lined ground squirrel (Current, 1989). Using “*Cryptosporidium*” and “squirrel” as key words during a recent literature search in PubMed conducted on January 13, 2015 resulted in only a few publications. *C. parvum* was reported in Eurasian red squirrels (*Sciurus vulgaris*) in Italy (Bertolino et al., 2003); *C. parvum* was also reported in Siberian chipmunks (*Tamias sibiricus*) originated from China and found infectious to SCID mice and ICR mice (Matsui et al., 2000); *Cryptosporidium muris* was reported in Siberian chipmunks (*Eutamias sibiricus*) imported from Southeast Asia to Czech Republic and found infectious to BALB/c mice (Hůrková et al., 2003); *Cryptosporidium* ferret genotype and chipmunk genotype were reported in red squirrels (*Sciurus vulgaris* L) in Italy and no detectable infection was found in CD1 mice and BALB/c mice after inoculation 1000 oocysts (Kvác et al., 2008). All these squirrel species belong to different genus other than *Spermophilus*. The only documentation of *Cryptosporidium* in *Spermophilus* genus besides ours was a report of *C. parvum* in spotted souslik (*Spermophilus suslicus*) in Poland (Kloch and Bajer, 2012). *S. suslicus* is a different species with distinct geographic distributions compared to *Spermophilus* ground squirrels in California, USA. In contrast to these sporadic detections of *Cryptosporidium* in different species of squirrels, we have consistently detected the *Cryptosporidium* sp. c-genotype in *Spermophilus* ground squirrels from throughout California over the past decade (Atwill et al., 2001, 2004; Pereira et al., 2010 present work).

Describing a novel species of *Cryptosporidium* requires four attributes to be satisfied: 1) genetic characterization; 2) morphometric studies of oocysts; 3) demonstration of natural and at least some experimental host specificity; and 4) compliance with International Commission on Zoological Nomenclature (ICZN) (Xiao et al., 2004; Fayer, 2010). Combining our current work and previous works (Atwill et al., 2004; Pereira et al., 2010), we have satisfied the requirements of genetic and morphometric characteristics as well as host specificity studies similar in scope to other researchers who have established *Cryptosporidium scrofarum* (Kváč et al., 2013), *Cryptosporidium viatorum* (Elwin et al., 2012), *Cryptosporidium xiaoi* (Fayer and Santín, 2009), *Cryptosporidium ryanae* (Fayer et al., 2008), *Cryptosporidium fayeri* (Ryan et al., 2008), and *Cryptosporidium bovis* (Fayer et al., 2005). To comply with ICZN, we provide morphological description of c-genotype oocysts (see above) and present DIC photos of Sbey11c oocysts from *S. beecheyi* collected in 2011 (Fig. 1).

## 5 Conclusion

Our current and previous work has demonstrated that *Spermophilus* ground squirrels are the hosts of a distinct *Cryptosporidium* sp. c-genotype. Based on the findings from these work, the c-genotype in *Spermophilus* ground squirrels is described as *C. rubeyi* n. sp. Further studies are warranted to understand the geographic distribution, environmental dissemination, and epidemiology including age and sex related prevalence of *C. rubeyi* n. sp. in *Spermophilus* ground squirrels.

## Conflicts of interest

The authors declared that there is no conflict of interest.

## Figures and Tables

**Table 1:** Morphology of *Cryptosporidium* sp. c-genotype oocysts from *Spermophilus* ground squirrels.

Squirrel species	Isolate		Oocyst size		
Squirrel ID	Genotype^a^	Length (μm) (mean ± SD)	Width (μm) (mean ± SD)	Shape index^b^ (mean ± SD)
*S. lateralis*	181	Sltl05c	4.74 ± 0.13	4.34 ± 0.13^d^	1.09 ± 0.04
113	Sltl05c	4.65 ± 0.14^d^	4.38 ± 0.11^d^	1.06 ± 0.04
127	Sltl05c	4.62 ± 0.14^d^	4.28 ± 0.15^d^	1.08 ± 0.04
128	Sltl05c	4.60 ± 0.15^d^	4.23 ± 0.18^d^	1.09 ± 0.04
155	Sltl05c	4.74 ± 0.13	4.36 ± 0.12^d^	1.09 ± 0.04
230	Sltl05c	4.66 ± 0.22^c^	4.36 ± 0.22^d^	1.09 ± 0.05
121	Sltl05c	4.73 ± 0.13	4.46 ± 0.09^d^	1.06 ± 0.03
185	Sltl05c	4.58 ± 0.16^d^	4.22 ± 0.16^d^	1.09 ± 0.04
Mean			4.67 ± 0.16	4.33 ± 0.17	1.08 ± 0.04
*S. beecheyi*	560	Sbey05c	4.68 ± 0.16^c^	4.39 ± 0.14^d^	1.07 ± 0.04
573	Sbey05c	4.70 ± 0.17	4.45 ± 0.14^d^	1.06 ± 0.04
Mean			4.69 ± 0.16	4.42 ± 0.14	1.06 ± 0.04
*S. beldingi*	329	Sbld05c	4.68 ± 0.16^c^	4.27 ± 0.26^d^	1.10 ± 0.07
Overall mean			4.67 ± 0.16	4.34 ± 0.18	1.08 ± 0.05
*C. parvum* bovine genotype			4.81 ± 0.19	4.66 ± 0.23	1.03 ± 0.04

a Genotypes from *Spermophilus* ground squirrels in California, USA, see Pereira et al., 2010 for reference.

b Shape index = ratio of length/width.

c 0.01 < p < 0.05 (compared to *C. parvum* bovine genotype oocysts).

d p < 0.01 (compared to *C. parvum* bovine genotype oocysts).

**Table 2:** BALB/c mice infectivity assay of *Cryptosporidium* sp. c-genotype oocysts from *Spermophilus* ground squirrels.

Squirrel species	Isolate		No. of infected pups/no. of inoculated pups at different doses of oocysts		
Squirrel ID	Genotype^a^	100	5000	10,000
*S. lateralis*	181	Sltl05c	0/6	0/6	ND
113	Sltl05c	0/7	0/6	0/6
127	Sltl05c	0/7	0/4	ND
128	Sltl05c	0/7	0/6	0/5
155	Sltl05c	0/7	0/6	0/8
230	Sltl05c	0/8	0/5	0/6
121	Sltl05c	0/7	0/5	ND
185	Sltl05c	0/6	0/6	ND
*S. beecheyi*	560	Sbey05c	0/7	0/6	0/8
	573	Sbey05c	0/8	0/5	0/3
*S. beldingi*	329	Sbld05c	ND^b^	ND	0/6
Positive control*-C. parvum* bovine genotype			5/6	19/19	17/17
Negative control-inactivated *C. parvum* bovine genotype			ND	ND	0/6
Negative control-mice inoculated with DI water			0/32		

a Genotypes from *Spermophilus* ground squirrels in California, USA, see Pereira et al., 2010 for reference.

b ND = not done.

**Table 3:** Gene sequences of *Cryptosporidium* sp. Sbey11c genotype^a^ isolated from *S. beecheyi* compared to *Cryptosporidium* isolates in the GenBank by BLAST analysis (conducted on March 11, 2015).

Gene	No. of isolates	Length (no. bp)	GenBank access no.	Closely related isolates and accession no. in the GenBank	Max. identity (%)
18S rRNA	18	830	KM010224	*Cryptosporidium.* sp. Sbey05c (DQ295012)	100
				*Cryptosporidium.* sp. Sbey03c (AY462233)	100
				*Cryptosporidium.* sp. Sltl05c (DQ295014)	99.64
				*Cryptosporidium.* sp. Sbld05c (DQ295013)	98.67
				*Cryptosporidium* environmental sequence isolate CRY1636 (JQ178292)	97.17
				*Cryptosporidium suis* isolate QP8 (JF710259)	97.00
Actin	14	996	KM010227	*Cryptosporidium* sp. chipmunk genotype I isolate Swec176 (JX978270)	93.20
HSP70	3	1792	KM010229	*Cryptosporidium* sp. chipmunk genotype I isolate Swec176 (JX978276)	92.35
				*Cryptosporidium* sp. chipmunk genotype I isolate Swec096 (JX978275)	92.29

a The *Cryptosporidium* sp. Sbey11c genotype was collected from multiple *S. beecheyi* hosts in 2011 from central coastal California.

## References

[bib1] Akiyoshi D.E., Feng X., Buckholt M.A., Widmer G., Tzipori S. (2002). Genetic analysis of a *Cryptosporidium parvum* human genotype 1 isolate passaged through different host species. Infect. Immun..

[bib2] Arrowood M.J., Sterling C.R. (1987). Isolation of *Cryptosporidium oocysts* and sporozoites using discontinuous sucrose and isopycnic Percoll gradients. J. Parasitol..

[bib3] Atwill E.R., Camargo S.M., Phillips R., Alonso L.H., Tate K.W., Jensen W.A., Bennet J., Little S., Salmon T.P. (2001). Quantitative shedding of two genotypes of *Cryptosporidium parvum* in California ground squirrels (*Spermophilus beecheyi*). Appl. Environ. Microbiol..

[bib4] Atwill E.R., Phillips R., Pereira M.D., Li X., McCowan B. (2004). Seasonal shedding of multiple *Cryptosporidium* genotypes in California ground squirrels (*Spermophilus beecheyi*). Appl. Environ. Microbiol..

[bib5] Atwill E.R., Pereira M.D., Alonso L.H., Elmi C., Epperson W.B., Smith R., Riggs W., Carpenter L.V., Dargatz D.A., Hoar B. (2006). Environmental load of *Cryptosporidium parvum* oocysts from cattle manure in feedlots from the central and western United States. J. Environ. Qual..

[bib6] Bertolino S., Wauters L.A., De Bruyn L., Canestri-Trotti G. (2003). Prevalence of coccidia parasites (Protozoa) in red squirrels (*Sciurus vulgaris*): effects of host phenotype and environmental factors. Oecologia.

[bib7] Boellstorff D.E., Owings D.H. (1995). Home range, population structure, and spatial organization of California ground squirrels. J. Mammol..

[bib8] Brook E.J., Anthony Hart C., French N.P., Christley R.M. (2009). Molecular epidemiology of *Cryptosporidium* subtypes in cattle in England. Vet. J..

[bib9] Chalmers R.M., Robinson G., Elwin K., Hadfield S.J., Thomas E., Watkins J., Casemore D., Kay D. (2010). Detection of *Cryptosporidium* species and sources of contamination with *Cryptosporidium hominis* during a waterborne outbreak in North West Wales. J. Water Health.

[bib10] Checkley W., White A.C., Jaganath D., Arrowood M.J., Chalmers R.M., Chen X., Fayer R., Griffiths J.K., Guerrant R.L., Hedstrom L., Huston C.D., Kotloff K.L., Kang G., Mead J.R., Miller M., Petri W.A., Priest J.W., Roos D.S., Striepen B., Thompson R.C., Ward H.D., Van Voorhis W.A., Xiao L., Zhu G., Houpt E.R. (2015). A review of the global burden, novel diagnostics, therapeutics, and vaccine targets for *Cryptosporidium*. Lancet Infect. Dis..

[bib11] Current W.L., Walzer P.D., Genta R.M. (1989). *Cryptosporidium* spp. Parasitic Infections in the Compromised Host.

[bib12] Elwin K., Hadfield S.J., Robinson G., Crouch N.D., Chalmers R.M. (2012). *Cryptosporidium viatorum* n. sp. (Apicomplexa: Cryptosporidiidae) among travellers returning to Great Britain from the Indian subcontinent, 2007–2011. Int. J. Parasitol..

[bib13] Fall A., Thompson R.C., Hobbs R.P., Morgan-Ryan U. (2003). Morphology is not a reliable tool for delineating species within *Cryptosporidium*. J. Parasitol..

[bib14] Fayer R. (1995). Effect of sodium hypochlorite exposure on infectivity of *Cryptosporidium parvum* oocysts for neonatal BALB/c mice. Appl. Environ. Microbiol..

[bib15] Fayer R. (2010). Taxonomy and species delimitation in *Cryptosporidium*. Exp. Parasitol..

[bib16] Fayer R., Morgan U., Upton S.J. (2000). Epidemiology of *Cryptosporidium*: transmission, detection and identification. Int. J. Parasitol..

[bib17] Fayer R., Santín M. (2009). *Cryptosporidium xiaoi* n. sp. (Apicomplexa: Cryptosporidiidae) in sheep (*Ovis aries*). Vet. Parasitol..

[bib18] Fayer R., Santín M., Trout J.M. (2008). *Cryptosporidium ryanae* n. sp. (Apicomplexa: Cryptosporidiidae) in cattle (*Bos taurus*). Vet. Parasitol..

[bib19] Fayer R., Santín M., Xiao L. (2005). *Cryptosporidium bovis* n. sp. (Apicomplexa: Cryptosporidiidae) in cattle (*Bos taurus*). J. Parasitol..

[bib20] Feltus D.C., Giddings C.W., Schneck B.L., Monson T., Warshauer D., McEvoy J.M. (2006). Evidence supporting zoonotic transmission of *Cryptosporidium spp*. in Wisconsin. J. Clin. Microbiol..

[bib21] Feng Y., Alderisio K.A., Yang W., Blancero L.A., Kuhne W.G., Nadareski C.A., Reid M., Xiao L. (2007). *Cryptosporidium* genotypes in wildlife from a New York watershed. Appl. Environ. Microbiol..

[bib22] Finch G.R., Daniels C.W., Black E.K., Schaefer F.W., Belosevic M. (1993). Dose response of *Cryptosporidium parvum* in outbred neonatal CD-1 mice. Appl. Environ. Microbiol..

[bib23] Guk S.M., Yong T.S., Park S.J., Park J.H., Chai J.Y. (2004). Genotype and animal infectivity of a human isolate of *Cryptosporidium parvum* in the Republic of Korea. Korean J. Parasitol..

[bib24] Hill N.J., Deane E.M., Power M.L. (2008). Prevalence and genetic characterization of *Cryptosporidium* isolates from common brushtail possums (*Trichosurus vulpecula*) adapted to urban settings. Appl. Environ. Microbiol..

[bib25] Hou L., Li X., Dunbar L., Moeller R., Palermo B., Atwill E.R. (2004). Neonatal mouse infectivity of intact *Cryptosporidium parvum* oocysts isolated after optimized in vitro excystation. Appl. Environ. Microbiol..

[bib26] Hůrková L., Hajdusek O., Modrý D. (2003). Natural infection of *Cryptosporidium muris* (Apicomplexa: Cryptosporiidae) in Siberian chipmunks. J. Wildl. Dis..

[bib27] Jenkins M., Trout J.M., Higgins J., Dorsch M., Veal D., Fayer R. (2003). Comparison of tests for viable and infectious *Cryptosporidium parvum* oocysts. Parasitol. Res..

[bib28] Jiang J., Alderisio K.A., Xiao L. (2005). Distribution of *Cryptosporidium* genotypes in storm event water samples from three watersheds in New York. Appl. Environ. Microbiol..

[bib29] Kloch A., Bajer A. (2012). Natural infections with *Cryptosporidium* in the endangered spotted souslik (*Spermophilus suslicus*). Acta Parasitol..

[bib30] Kvác M., Hofmannová L., Bertolino S., Wauters L., Tosi G., Modrý D. (2008). Natural infection with two genotypes of *Cryptosporidium* in red squirrels (*Sciurus vulgaris*) in Italy. Folia Parasitol..

[bib31] Kváč M., Kestřánová M., Pinková M., Květoňová D., Kalinová J., Wagnerová P., Kotková M., Vítovec J., Ditrich O., McEvoy J., Stenger B., Sak B. (2013). *Cryptosporidium scrofarum* n. sp. (Apicomplexa: Cryptosporidiidae) in domestic pigs (*Sus scrofa*). Vet. Parasitol..

[bib32] Li X., Atwill E.R., Dunbar L.A., Jones T., Hook J., Tate K.W. (2005). Seasonal temperature fluctuations induce rapid inactivation of *Cryptosporidium parvum*. Environ. Sci. Technol..

[bib33] Li X., Atwill E.R., Dunbar L.A., Tate K.W. (2010). Effect of daily temperature fluctuation during the cool season on the infectivity of *Cryptosporidium parvum*. Appl. Environ. Microbiol..

[bib34] MacKenzie W.R., Hoxie N.J., Proctor M.E., Gradus M.S., Blair K.A., Peterson D.E., Kazmierczak J.J., Addiss D.G., Fox K.R., Rose J.B., Davis J.P. (1994). A massive outbreak in Milwaukee of *Cryptosporidium* infection transmitted through the public water supply. N. Engl. J. Med..

[bib35] Matsui T., Fujino T., Kajima J., Tsuji M. (2000). Infectivity to experimental rodents of *Cryptosporidium parvum* oocysts from Siberian chipmunks (*Tamias sibiricus*) originated in the People's Republic of China. J. Vet. Med. Sci..

[bib36] Matsui T., Fujino T., Tsuji M. (1999). Infectivity to hosts of the endogenous stages of chicken and murine *Cryptosporidium*. J. Vet. Med. Sci..

[bib37] Neumann N.F., Gyuerek L.L., Finch G.R., Belosevic M. (2000). Intact *Cryptosporidium parvum* oocysts isolated after in vitro excystation are infectious to neonatal mice. FEMS Microbiol. Lett..

[bib38] Owings D.H., Borchert M., Virginia R. (1977). The behavior of California ground squirrels. Anim. Behav..

[bib39] Pereira M.D., Li X., McCowan B., Phillips R.L., Atwill E.R. (2010). Multiple unique *Cryptosporidium* isolates from three species of ground squirrels (*Spermophilus beecheyi*, *S. beldingi* and *S. lateralis*) in California. Appl. Environ. Microbiol..

[bib40] Putignani L., Menichella D. (2010). Global distribution, public health and clinical impact of the protozoan pathogen *Cryptosporidium*. Interdiscip. Perspect. Infect. Dis..

[bib41] Raskova V., Kvetonova D., Sak B., McEvoy J., Edwinson A., Stenger B., Kvac M. (2013). Human cryptosporidiosis caused by *Cryptosporidium tyzzeri* and *C. parvum* isolates presumably transmitted from wild mice. J. Clin. Microbiol..

[bib42] Rhee J.K., So W.S., Kim H.C. (1999). Age-dependent resistance to *Cryptosporidium muris* (strain MCR) infection in golden hamsters and mice. Korean J. Parasitol..

[bib43] Ruecker N.J., Braithwaite S.L., Topp E., Edge T., Lapen D.R., Wilkes G., Robertson W., Medeiros D., Sensen C.W., Neumann N.F. (2007). Tracking host sources of *Cryptosporidium spp*. in raw water for improved health risk assessment. Appl. Environ. Microbiol..

[bib44] Ruecker N.J., Hoffman R.M., Chalmers R.M., Neumann N.F. (2011). Detection and resolution of *Cryptosporidium* species and species mixtures by genus-specific nested PCR-restriction fragment length polymorphism analysis, direct sequencing, and cloning. Appl. Environ. Microbiol..

[bib45] Ryan U.M., Power M., Xiao L. (2008). *Cryptosporidium fayeri* n. sp. (Apicomplexa: Cryptosporidiidae) from the Red Kangaroo (*Macropus rufus*). J. Eukaryot. Microbiol..

[bib46] Šlapeta J. (2013). Cryptosporidiosis and *Cryptosporidium* species in animals and humans: a thirty colour rainbow?. Int. J. Parasitol..

[bib47] Slifko T.R., Huffman D.E., Dussert B., Owens J.H., Jakubowski W., Haas C.N., Rose J.B. (2002). Comparison of tissue culture and animal models for assessment of *Cryptosporidium parvum* infection. Exp. Parasitol..

[bib48] Sulaiman I.M., Lal A.A., Xiao L. (2002). Molecular phylogeny and evolutionary relationships of *Cryptosporidium* parasites at the actin locus. J. Parasitol..

[bib49] Sulaiman I.M., Morgan U.M., Thompson R.C., Lal A.A., Xiao L. (2000). Phylogenetic relationships of *Cryptosporidium* parasites based on the 70- kilodalton heat shock protein (HSP70) gene. Appl. Environ. Microbiol..

[bib50] Sundberg J.P., Ryan M.J. (1982). *Cryptosporidium* in a gray squirrel. JAVMA.

[bib51] Tarazona R., Blewett D.A., Carmona M.D. (1998). *Cryptosporidium parvum* infection in experimentally infected mice: infection dynamics and effect of immunosuppression. Folia Parasitol. Praha.

[bib52] Xiao L., Alderisio K., Limor J., Royer M., Lal A.A. (2000). Identification of species and sources of *Cryptosporidium* oocysts in storm waters with a small-subunit rRNA-based diagnostic and genotyping tool. Appl. Environ. Microbiol..

[bib53] Xiao L., Fayer R., Ryan U., Upton S.J. (2004). *Cryptosporidium* taxonomy: recent advances and implications for public health. Clin. Microbiol. Rev..

[bib54] Xiao L., Ryan U.M. (2004). Cryptosporidiosis: an update in molecular epidemiology. Curr. Opin. Infec. Dis..

[bib55] Yang S., Benson S.K., Du C., Healey M.C. (2000). Infection of immunosuppressed C57BL/6N adult mice with a single oocyst of *Cryptosporidium parvum*. J. Parasitol..

[bib56] Youssef M.M., Amin S.M., Abou Samra L.M., el-Gebaly W.M., Hammam S.M., el-Sabaawy E., Khalifa A.M. (1992). A study on experimental cryptosporidiosis. J. Egypt Soc. Parasitol..

[bib57] Zhou L., Yang C., Xiao L. (2003). PCR-mediated recombination between *Cryptosporidium* spp. of lizards and snakes. J. Eukaryot. Microbiol..

